# Antihypertensive medication persistence and adherence among non-Hispanic Asian US patients with hypertension and fee-for-service Medicare health insurance

**DOI:** 10.1371/journal.pone.0300372

**Published:** 2024-03-20

**Authors:** Eunhee Choi, Hiroyuki Mizuno, Zhixin Wang, Chloe Fang, Matthew T. Mefford, Kristi Reynolds, Lama Ghazi, Daichi Shimbo, Paul Muntner

**Affiliations:** 1 The Columbia Hypertension Center and Lab, Columbia University Irving Medical Center, New York, New York, United Kingdom; 2 Division of Cardiovascular Medicine, Jichi Medical University School of Medicine, Tochigi, Japan; 3 Department of Epidemiology, School of Public Health, University of Alabama at Birmingham, Birmingham, Alabama, United States of America; 4 Department of Research and Evaluation, Kaiser Permanente of Southern California, Pasadena, California, United States of America; 5 Department of Health Systems Science, Kaiser Permanente Bernard J Tyson School of Medicine, Pasadena, California, United States of America; Chiesi USA, UNITED STATES

## Abstract

**Background:**

Less than 50% of non-Hispanic Asian adults taking antihypertensive medication have controlled blood pressure.

**Methods:**

We compared non-persistence and low adherence to antihypertensive medication between non-Hispanic Asian and other race/ethnicity groups among US adults ≥66 years who initiated antihypertensive medication between 2011 and 2018 using a 5% random sample of Medicare beneficiaries (non-Hispanic Asian, n = 2,260; non-Hispanic White, n = 56,000; non-Hispanic Black, n = 5,792; Hispanic, n = 4,212; and Other, n = 1,423). Non-persistence was defined as not having antihypertensive medication available to take in the last 90 of 365 days following treatment initiation. Low adherence was defined as having antihypertensive medication available to take on <80% of the 365 days following initiation.

**Results:**

In 2011–2012, 2013–2014, 2015–2016 and 2017–2018, the proportion of non-Hispanic Asian Medicare beneficiaries with non-persistence was 29.1%, 25.6%, 25.4% and 26.7% (p-trend = 0.381), respectively, and the proportion with low adherence was 58.1%, 54.2%, 53.4% and 51.6%, respectively (p-trend = 0.020). In 2017–2018, compared with non-Hispanic Asian beneficiaries, non-persistence was less common among non-Hispanic White beneficiaries (risk ratio 0.74 [95%CI, 0.64–0.85]), non-Hispanic Black beneficiaries (0.80 [95%CI 0.68–0.94]) and those reporting Other race/ethnicity (0.68 [95%CI, 0.54–0.85]) but not among Hispanic beneficiaries (1.04 [95%CI, 0.88–1.23]). Compared to non-Hispanic Asian beneficiaries, non-Hispanic White beneficiaries and beneficiaries reporting Other race/ethnicity were less likely to have low adherence to antihypertensive medication (relative risk 0.78 [95%CI 0.72–0.84] and 0.84 [95%CI 0.74–0.95], respectively); there was no association for non-Hispanic Black or Hispanic beneficiaries.

**Conclusions:**

Non-persistence and low adherence to antihypertensive medication were more common among older non-Hispanic Asian than non-Hispanic White adults.

## Introduction

Non-Hispanic Asians comprise 6.0% of the United States (US) population, representing 23.9 million Americans in 2020 [1]. Additionally, non-Hispanic Asian is the fastest-growing racial group in the US [1]. Data from the National Health and Nutrition Examination Survey (NHANES) indicate that the prevalence of hypertension among non-Hispanic Asian adults increased from 27.0% to 33.5% between 2009–2012 and 2017–2020, while the proportion of those taking antihypertensive medication that had controlled blood pressure (BP) did not change over this period [2]. In 2017–2020, 45.5% of non-Hispanic Asian US adults with hypertension had controlled BP, defined as systolic BP < 140 mm Hg and diastolic BP < 90 mm Hg.

BP control has been recognized as a health equity issue, and preventing hypertension-related cardiovascular disease (CVD) requires increasing the proportion of all race/ethnic groups with controlled BP [3]. Non-persistence, defined as discontinuing treatment, and low adherence, defined as not taking treatment as prescribed, to antihypertensive medication are major barriers to achieving BP control [4–6]. Several domains of influence in the National Institute of Minority Health and Health Disparities Research Framework including behavioral, sociocultural and health care system factors contribute to non-persistence and low adherence to antihypertensive medication [7–10]. A 2021 working group report from the National Heart, Lung, and Blood Institute emphasized the limited data available for non-Hispanic Asian US adults [11]. Estimating trends in non-persistence and low adherence to antihypertensive medication among non-Hispanic Asians, and the factors associated with non-persistence and low adherence, can inform interventions to improve BP control.

For the current study, we estimated changes between 2011–2012 and 2017–2018 in the proportion of non-Hispanic Asian Medicare beneficiaries with non-persistence and low adherence to antihypertensive medication in the year following treatment initiation. To provide context for these results, we compared the proportion of non-Hispanic Asian Medicare beneficiaries with non-persistence and low adherence to antihypertensive medication to the proportions for other race/ethnicity groups. In addition, we identified demographic characteristics and comorbid conditions associated with non-persistence and low adherence among non-Hispanic Asians and people in other race/ethnicity groups.

## Material and methods

We conducted a retrospective cohort study of older US adults who initiated antihypertensive medication between 2011 and 2018 using a 5% random sample of Medicare beneficiaries. Medicare is a US government health insurance program for adults ≥ 65 years of age and younger adults who are disabled or with end-stage kidney disease. For each Medicare beneficiary, we obtained de-identified data on inpatient and outpatient medical services and outpatient prescription claims. The data were accessed for research purposes on 12/07/2021. The authors did not have access to information that could identify individual participants during or after data analysis (i.e. data were de-identified). The use of these data was approved by the University of Alabama at Birmingham Institutional Review Board. A waiver of informed consent was granted for the use of the de-identified data. Medicare data to replicate this analysis are not publicly available but can be licensed for a fee through the Research Data Assistance Center (https://resdac.org/research-identifiable-files-rif-requests). Statistical code used for this analysis is available as a supplemental item.

We included Medicare beneficiaries with a single race or ethnicity, determined using the Social Security Administration and a Centers for Medicare and Medicaid Services algorithm [12]. Beneficiaries were required to fill one or more prescriptions for antihypertensive medication between January 1, 2011 and December 31, 2018 (**S1 Table in the Online Supplement**). For each beneficiary, the first claim for an antihypertensive medication fill in each calendar year was identified. The date of this fill was defined as the index date for that year. Beneficiaries were excluded if they had inconsistencies in their administrative data (e.g., more than one date of birth). Beneficiaries whose age was ≥ 66 years on their index date were included. We restricted the analyses to Medicare beneficiaries with continuous inpatient, outpatient, and pharmacy coverage (Medicare Parts A, B, and D, respectively) from 365 days before their index date (i.e., the look-back period) through 365 days following their index date (i.e., the follow-up period). Beneficiaries with Medicare advantage (Part C coverage), a capitated program for which complete pharmacy claims were not available, at any point in the look-back or follow-up period were excluded from the analyses. Beneficiaries were excluded if they lived outside of the US during the look-back or follow-up periods, or if they died before the end of the follow-up period. The analyses were further restricted to beneficiaries with a history of hypertension defined by one or more outpatient physician evaluation and management claim during the look-back period with International Classification of Diseases, 9th Revision [ICD-9] diagnoses of 401, 402, 403 or 404 until October 2015 or 10th Revision [ICD-10] codes of I10, I11, I12, or I13 during or after October 2015. Beneficiaries were excluded if they filled an antihypertensive medication during their look-back period defined by the presence of a pharmacy claim. Beneficiaries were included in each calendar year they met the inclusion criteria. Overall, 69,687 beneficiaries were included in the analysis with a total number of 76,039 records (i.e., 6,352 beneficiaries were included in more than one calendar year).

### Antihypertensive medication fills

Antihypertensive medication fills were based on outpatient prescription claims and included angiotensin-converting enzyme (ACE) inhibitors, angiotensin receptor blockers (ARB), β-blockers, calcium channel blockers, loop diuretics, thiazide diuretics, and other classes that were grouped together. The other classes included aldosterone receptor antagonists, α-blockers, central acting agents, direct vasodilators, potassium-sparing diuretics, and renin inhibitors. The regimen initiated by each patient was defined based on all antihypertensive medications they filled on their index date and in the next 7 days. The antihypertensive medication regimen was categorized as being a single class, multiple classes with the use of multiple pills, or fixed-dose combination therapy. Fixed-dose combination therapy was defined as a single pill containing 2 or more antihypertensive medication classes. If a patient filled a fixed-dose combination medication and an additional antihypertensive medication in another pill, the patient was categorized as taking fixed-dose combination therapy.

### Outcomes

**Fig 1:**
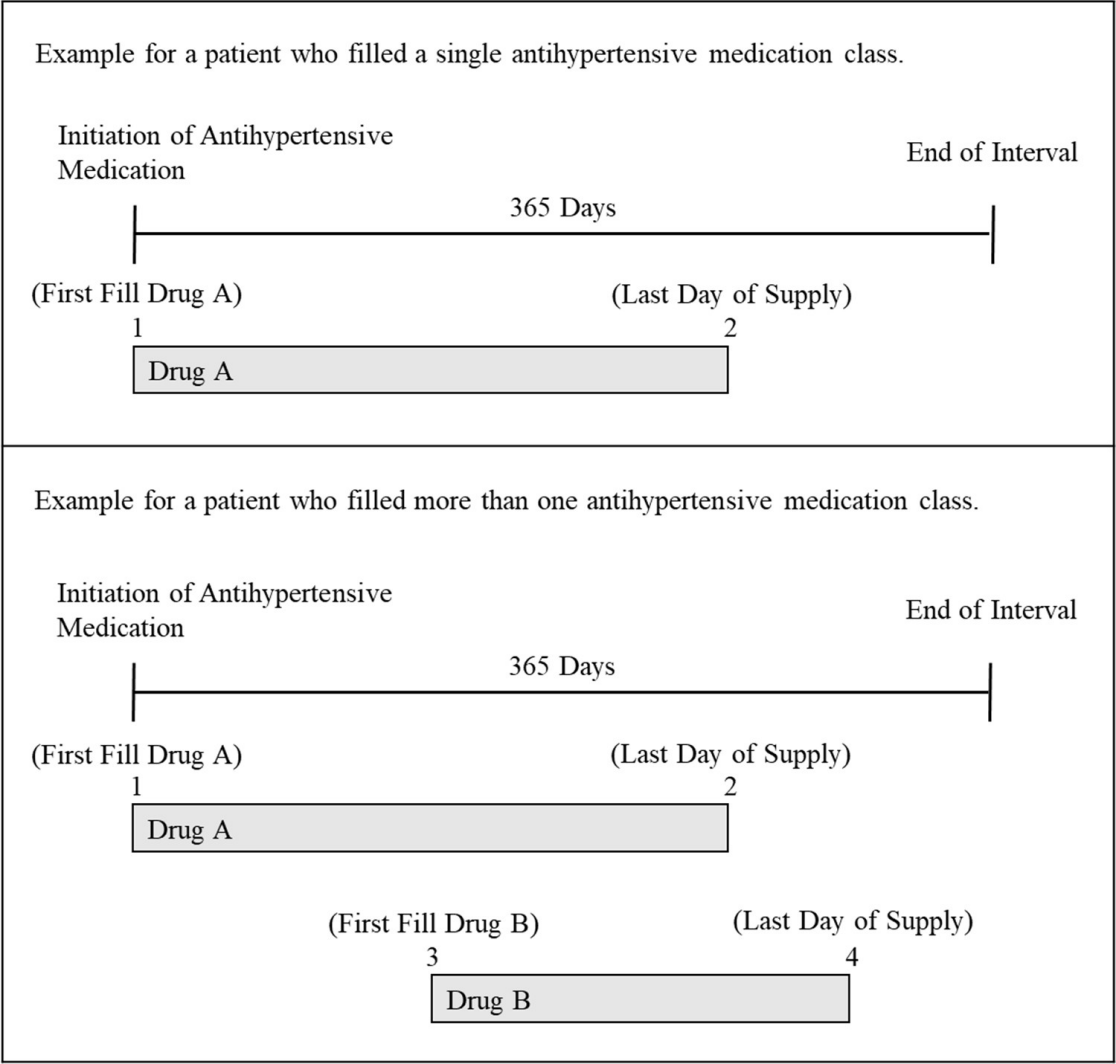
Interval-based proportion of days covered (PDC) calculation method for defining low adherence to antihypertensive medication. The gray boxes indicate the days on which supply of antihypertensive medication is available to take. For beneficiaries who filled one antihypertensive medication class during the 365 days follow-up period (top panel), the PDC was calculated using the number of days on which antihypertensive medication was available to take (i.e., from 1 to 2) during the 365 days follow-up period as the numerator and the numbers of days not spent hospitalized in the 365 days follow-up period as the denominator. For beneficiaries who filled more than one antihypertensive medication class during the 365 days follow-up period (bottom panel), the PDC was calculated by counting days on which any antihypertensive medication was available (i.e., from 1 to 4) to take as the numerator. Abbreviations: PDC, proportion of days covered.

The two primary outcomes were non-persistence and low adherence to antihypertensive medication in the 365 days following treatment initiation (i.e., the follow-up period). A secondary outcome was low adherence among those with persistence to antihypertensive medication in the follow-up period. Non-persistence and persistence were defined as not having and having antihypertensive medication available to take, respectively, in the last 90 days of the follow-up period regardless of the number of classes of antihypertensive medication in the patient’s initial regimen [6]. Antihypertensive medication adherence was calculated using the interval-based proportion of days covered (PDC) method [13]. The PDC was calculated using the number of days on which any antihypertensive medication was available to take during the follow-up period as the numerator and the numbers of days not spent hospitalized in the follow-up period as the denominator (**Fig 1, top panel**) [13, 14]. As patients are typically provided medication while hospitalized, days spent in the hospital were not counted in the numerator or denominator. For beneficiaries whose initiation regimen included more than one antihypertensive medication class or those who filled more than one antihypertensive medication class during the 365 days following initiation, the PDC was calculated by counting days on which any antihypertensive medication was available to take as the numerator (**Fig 1, bottom panel**). Low adherence to antihypertensive medication was defined by a PDC <80% [4, 13, 15]. A PDC for antihypertensive medication <80% has been shown to be associated with an increased risk for CVD events [16].

### Other characteristics of Medicare beneficiaries

Age, race/ethnicity, sex, and residence in the US were obtained from the Medicare beneficiary summary file. Race/ethnicity was categorized into five groups: non-Hispanic Asian, non-Hispanic White, non-Hispanic Black, Hispanic and Other (e.g., American Indian/Alaska Native). Using previously published algorithms [17–23], we identified whether patients had diabetes, CVD, heart failure, chronic kidney disease (CKD), depression, and a serious fall injury [24]. When identified during the look-back period, these were categorized as being prevalent conditions. For patients without these conditions during the look-back period, the conditions were considered to be newly documented if they were identified using the claims algorithms during the follow-up period. Polypharmacy was defined as having claims for ≥ 10 different medication classes during the look-back period. We calculated each patient’s copay-per-day of supply for their initial antihypertensive medication treatment regimen. Patients having one or more days without pharmacy coverage in the follow-up period were considered to have a Medicare Part D coverage gap, due to spending more than the maximal limit on what the plan will cover but not reaching the spending amount when Medicare Catastrophic Coverage begins to pay for prescription medications.

### Statistical analyses

To provide more stable statistical estimates, we combined years into two-year calendar periods for all analyses. Specifically, beneficiaries were grouped as having initiated antihypertensive medication in 2011–2012, 2013–2014, 2015–2016, and 2017–2018. We summarized beneficiary characteristics by calendar period and race/ethnicity. The proportion of all beneficiaries initiating antihypertensive medication with non-persistence and low adherence, separately, and the proportion with low adherence among those who were persistent were calculated by calendar period and race/ethnicity. We conducted a Mantel-Haenszel test for linear trend across calendar periods within each race/ethnicity group. Using Poisson regression models with robust variances estimators, we estimated the adjusted risk ratios (RRs) with 95% confidence intervals (95%CI) for having non-persistence and low adherence associated with each race/ethnicity group (non-Hispanic White, non-Hispanic Black, Hispanic, and other) versus non-Hispanic Asian beneficiaries as the reference group for the overall period (2011–2018) and the most recent calendar period (2017–2018). Poisson regression can be used to model binary outcomes and provide valid risk estimates and confidence levels with appropriate methods [25]. Low adherence was evaluated among all Medicare beneficiaries who initiated antihypertensive medication and among those who were persistent to their antihypertensive medication. Variables included in the model were age (66 to 74, 75 to 84, and ≥85 years), sex, antihypertensive medication class initiated during the follow-up period (ACE inhibitor, angiotensin receptor blocker, beta blocker, calcium channel blocker, loop diuretic, thiazide diuretic, and other medication), antihypertensive medication regimen initiated during the follow-up period (single class, multiple classes with multiple pills, and fixed-dosed combination therapy), initiation with a 90-day fill, copay-per-day of supply, prevalent conditions including diabetes, CVD, heart failure, CKD, depression, serious fall injury, polypharmacy, newly documented conditions including diabetes, CKD, CVD, depression, serious fall injury, and having a Medicare Part D coverage gap. Additionally, calendar period was included as a covariate in the analysis of the overall period. The proportion with, and relative risk for, non-persistence and low adherence were also calculated for women and men, separately.

Several sensitivity analyses were performed. First, using the same approach described above, we estimated the proportion with, and relative risk for, non-persistence and low adherence using three separate definitions of non-persistence: (1) not having medication available to take for ≥90 consecutive days at any time during the follow-up period, (2) not having medication available to take during the last 60 days of the follow-up period, and (3) not having medication available to take for ≥60 consecutive days at any time during the follow-up period. Second, we estimated the median, 25^th^ and 75^th^ percentiles of PDC for non-Hispanic Asian, non-Hispanic White, non-Hispanic Black, Hispanic, and other beneficiaries in each calendar period. Using quantile regression, we estimated the difference (95% confidence interval) in median PDC for each race/ethnicity group (non-Hispanic White, non-Hispanic Black, Hispanic, and those reporting other race/ethnicity) versus non-Hispanic Asian beneficiaries with adjustment as described above.

For each race/ethnicity group, using the overall period, we estimated the adjusted RRs for having non-persistence and low adherence associated with these variables in the multivariable-adjusted models. A p-value less than 0.05 was considered statistically significant, and all statistical analyses were conducted using SAS software version 9.4 (SAS Institute, Cary, NC, USA).

## Results

### Beneficiary characteristics

There were 2,260 non-Hispanic Asian beneficiaries, 56,000 non-Hispanic White beneficiaries, 5,792 non-Hispanic Black beneficiaries, 4,212 Hispanic beneficiaries and 1,423 beneficiaries with Other race/ethnicity included in the current analyses (**Table 1** and **S2 Table**). Among the non-Hispanic Asian beneficiaries initiating antihypertensive medication, 48.6% were 66 to 74 years old, 57.7% were female, and 81.3%, 10.0%, and 8.7% initiated antihypertensive medication with a single class, multiple classes with multiple pills, and fixed-dose combination therapy, respectively.

**Table 1:** Characteristics of Medicare beneficiaries initiating antihypertensive medication by race/ethnicity.

	Race/ethnicity				
	Non-Hispanic Asian (n = 2,260)	Non-Hispanic White (n = 56,000)	Non-Hispanic Black (n = 5,792)	Hispanic (n = 4,212)	Other (n = 1,423)
Calendar year of initiation					
2011–2012	623 (27.6%)	14,903 (26.6%)	1,585 (27.4%)	1,285 (30.5%)	276 (19.4%)
2013–2014	542 (24.0%)	13,727 (24.5%)	1,489 (25.7%)	1,026 (24.4%)	276 (19.4%)
2015–2016	497 (22.0%)	13,240 (23.6%)	1,313 (22.7%)	925 (22.0%)	391 (27.5%)
2017–2018	598 (26.5%)	14,130 (25.2%)	1,405 (24.3%)	976 (23.2%)	480 (33.7%)
Age, years					
66–74	1,098 (48.6%)	26,663 (47.6%)	3,197 (55.2%)	2,193 (52.1%)	967 (68.0%)
75–84	843 (37.3%)	19,654 (35.1%)	1,766 (30.5%)	1,424 (33.8%)	347 (24.4%)
85+	319 (14.1%)	9,683 (17.3%)	829 (14.3%)	595 (14.1%)	109 (7.7%)
Female sex	1,303 (57.7%)	33,290 (59.4%)	3,466 (59.8%)	2,506 (59.5%)	722 (50.7%)
Antihypertensive medication class					
Thiazide diuretic	283 (12.5%)	8,769 (15.7%)	1,338 (23.1%)	697 (16.5%)	215 (15.1%)
ACE inhibitor	515 (22.8%)	17,812 (31.8%)	1,700 (29.4%)	1,645 (39.1%)	497 (34.9%)
Angiotensin receptor blocker	702 (31.1%)	8,105 (14.5%)	886 (15.3%)	767 (18.2%)	286 (20.1%)
Calcium Channel blocker	573 (25.4%)	9,849 (17.6%)	1,810 (31.3%)	815 (19.3%)	286 (20.1%)
Beta blocker	395 (17.5%)	12,597 (22.5%)	1,015 (17.5%)	720 (17.1%)	268 (18.8%)
Loop diuretic	101 (4.5%)	6,395 (11.4%)	610 (10.5%)	339 (8.0%)	108 (7.6%)
Other	172 (7.6%)	5,793 (10.3%)	872 (15.1%)	366 (8.7%)	144 (10.1%)
Antihypertensive medication pills					
Single class	1,837 (81.3%)	44,690 (79.8%)	3,950 (68.2%)	3,262 (77.4%)	1,118 (78.6%)
Multiple classes with multiple Pills	227 (10.0%)	6,489 (11.6%)	1,087 (18.8%)	512 (12.2%)	203 (14.3%)
Fix-dosed combination therapy*	196 (8.7%)	4,821 (8.6%)	755 (13.0%)	438 (10.4%)	102 (7.2%)
Initiated with a 90-day fill	911 (40.3%)	21,084 (37.7%)	1,757 (30.3%)	1,397 (33.2%)	547 (38.4%)
Copay-per-day of supply, $	0.1 (0.4)	0.2 (0.4)	0.1 (0.4)	0.1 (0.3)	0.1 (0.3)
Copay-per-day of supply, $					
Quartile 1 (< $0.0334)	789 (34.9%)	12,909 (23.1%)	1,794 (31.0%)	1,550 (36.8%)	450 (31.6%)
Quartile 2 ($0.0334- $0.0889	694 (30.7%)	12,861 (23.0%)	1,880 (32.5%)	1,581 (37.5%)	425 (29.9%)
Quartile 3 ($0.0890 - $0.1690)	436 (19.3%)	14,942 (26.7%)	1,024 (17.7%)	590 (14.0%)	280 (19.7%)
Quartile 4 (> $0.1690)	341 (15.1%)	15,288 (27.3%)	1,094 (18.9%)	491 (11.7%)	268 (18.8%)
Prevalent conditions					
Diabetes	781 (34.6%)	14,354 (25.6%)	2,122 (36.6%)	1,775 (42.1%)	475 (33.4%)
CVD	544 (24.1%)	16,760 (29.9%)	1,513 (26.1%)	1,112 (26.4%)	341 (24.0%)
Heart failure	82 (3.6%)	3,859 (6.9%)	544 (9.4%)	266 (6.3%)	71 (5.0%)
CKD	384 (17.0%)	9,345 (16.7%)	1,391 (24.0%)	735 (17.5%)	233 (16.4%)
Depression	311 (13.8%)	16,089 (28.7%)	1,000 (17.3%)	1,002 (23.8%)	308 (21.6%)
Serious fall injury	44 (1.9%)	1,812 (3.2%)	77 (1.3%)	105 (2.5%)	24 (1.7%)
Polypharmacy	740 (32.7%)	20,159 (36.0%)	1,461 (25.2%)	1,632 (38.7%)	429 (30.1%)
Following treatment initiation					
Newly documented diabetes	38 (1.7%)	618 (1.1%)	116 (2.0%)	70 (1.7%)	16 (1.1%)
Newly documented CKD	76 (3.4%)	2,113 (3.8%)	272 (4.7%)	156 (3.7%)	45 (3.2%)
Newly documented CVD	83 (3.7%)	2,222 (4.0%)	249 (4.3%)	206 (4.9%)	49 (3.4%)
Newly documented depression	66 (2.9%)	2,430 (4.3%)	256 (4.4%)	195 (4.6%)	69 (4.8%)
Serious fall injury	9 (0.4%)	555 (1.0%)	22 (0.4%)	32 (0.8%)	6 (0.4%)
Medicare Part D coverage gap	584 (25.8%)	11,942 (21.3%)	1,021 (17.6%)	1,076 (25.5%)	297 (20.9%)

Data are expressed as mean (SD) for copay-for-day of supply and number (percent) for other characteristics.

*Fixed-dose combination therapy is defined as initiating treatment with a single pill containing 2 or more antihypertensive classes. If a patient was prescribed fixed-dose combination therapy and an additional antihypertensive medication in another pill, the patient was categorized as taking fixed-dose combination therapy.

Abbreviations: ACE, angiotensin-converting enzyme; CVD, cardiovascular disease; CKD, chronic kidney disease.

### Non-persistence and low adherence

There was no evidence of a trend in non-persistence from 2011–2012 to 2017–2018 among non-Hispanic Asian, non-Hispanic Black, Hispanic, and beneficiaries of Other race/ethnicity groups (**Table 2, top panel**). The proportion of non-Hispanic White beneficiaries with non-persistence increased from 21.1% to 22.8% between 2011–2012 and 2017–2018. From 2011–2012 to 2017–2018, the proportion of beneficiaries who initiated antihypertensive medication with low adherence declined from 58.1% to 51.6% among non-Hispanic Asians, (p-trend = 0.020), from 45.9% to 44.2% among non-Hispanic Whites (p-trend = 0.006), from 62.6% to 57.6% among Hispanics (p-trend = 0.009), and from 51.8% to 46.1% among beneficiaries of Other race/ethnicity groups (p-trend = 0.069) (**Table 2, middle panel**). Among non-Hispanic Black beneficiaries, 58.9% and 57.1% had low adherence in 2011–2012 and 2017–2018, respectively (p-trend = 0.757). The proportion of patients with low adherence among those who were persistent is shown in the bottom panel of **Table 2.** The prevalence of non-persistence and low adherence for each race/ethnicity group further stratified by sex is presented in **S3 Table**.

**Table 2:** Race/ethnicity-specific proportion of beneficiaries with non-persistence and low adherence who initiated antihypertensive medication and low adherence among those who were persistent by two-year calendar periods.

	Race/ethnicity				
	Non-Hispanic Asian	Non-Hispanic White	Non-Hispanic Black	Hispanic	Other
Non-persistence, n (%)					
2011–2012	181 (29.1%)	3,149 (21.1%)***	401 (25.3%)	365 (28.4%)	62 (22.5%)*
2013–2014	149 (25.6%)	3,137 (21.4%)*	370 (23.2%)	308 (28.0%)	59 (20.3%)
2015–2016	146 (25.4%)	3,297 (22.4%)	413 (27.5%)	270 (25.6%)	81 (18.9%)*
2017–2018	185 (26.7%)	3,668 (22.8%)*	375 (23.6%)	320 (29.0%)	106 (20.1%)**
P-trend	0.381	<0.001	0.870	0.889	0.451
Low adherence among all beneficiaries who initiated antihypertensive medication, n (%)					
2011–2012	362 (58.1%)	6,842 (45.9%)***	934 (58.9%)	804 (62.6%)	143 (51.8%)
2013–2014	316 (54.2%)	6,634 (45.2%)***	883 (55.3%)	650 (59.0%)	149 (51.2%)
2015–2016	307 (53.4%)	6,688 (45.4%)***	888 (59.2%)*	606 (57.5%)	201 (47.0%)*
2017–2018	357 (51.6%)	7,103 (44.2%)***	906 (57.1%)*	637 (57.6%)*	243 (46.1%)
P-trend	0.020	0.006	0.757	0.009	0.069
Low adherence among those who had persistence, n (%)					
2011–2012	181 (41.0%)	3,694 (31.4%)***	533 (45.0%)	439 (47.7%)*	81 (37.9%)
2013–2014	167 (38.5%)	3,497 (30.3%)***	513 (41.8%)	342 (43.1%)	90 (38.8%)
2015–2016	161 (37.5%)	3,391 (29.6%)***	475 (43.7%)*	336 (42.9%)	120 (34.6%)
2017–2018	172 (33.9%)	3,435 (27.7%)**	531 (43.8%)***	317 (40.4%)*	137 (32.5%)
P-trend	0.026	<0.001	0.794	0.003	0.092

Data are expressed as number (percent) for outcomes. P-trend represents the trend across the calendar periods.

* p-value of 0.01 to 0.05 compared to non-Hispanic Asians within each calendar period

** p-value of 0.001 to 0.01 compared to non-Hispanic Asians within each calendar period

*** p-value <0.001 compared to non-Hispanic Asians within each calendar period.

### Multivariable-adjusted risk of non-persistence and low adherence

After multivariable adjustment, non-Hispanic White, non-Hispanic Black, and beneficiaries with Other race/ethnicity were each less likely than non-Hispanic Asian beneficiaries to have non-persistence to antihypertensive medication in both the overall period, 2011–2018, and in the most recent time period, 2017–2018 (**Table 3**). Among all patients who initiated antihypertensive medication and those who were persistent, compared with non-Hispanic Asian beneficiaries, non-Hispanic White beneficiaries were less likely to have low adherence after multivariable adjustment in 2011–2018 and 2017–2018. Among those with persistence, non-Hispanic Black patients were more likely than non-Hispanic Asian patients to have low adherence in 2011–2018 and 2017–2018. Across the entire period studied, 2011–2018, Hispanic patients were more likely than non-Hispanic Asians to have low adherence in the overall population and among those who were persistent. In 2017–2018, after multivariable adjustment, there was no evidence of a difference in low adherence in the overall population and among those who were persistent between non-Hispanic Asian and Hispanic patients. Among all patients who initiated antihypertensive medication, those of Other race/ethnicity were less likely to have low adherence compared with non-Hispanic Asian beneficiaries. Multivariable-adjusted risk ratios for non-persistence and low adherence for each race/ethnicity group compared with non-Hispanic Asian patients stratified by sex is presented in **S4 Table**.

**Table 3:** Multivariable-adjusted risk ratios (95% CI) for non-persistence and low adherence among beneficiaries who initiated antihypertensive medication and low adherence among beneficiaries with persistence in 2011–2018 and 2017–2018.

	Adjusted risk ratio (95% confidence interval)		
	Non-persistence N = 69,687	Low adherence, overall population N = 69,687	Low adherence, among those with persistence N = 54,199
	2011–2018		
Non-Hispanic Asian	1 (ref)	1 (ref)	1 (ref)
Non-Hispanic White	0.74 (0.69–0.80)	0.78 (0.75–0.81)	0.74 (0.70–0.79)
Non-Hispanic Black	0.88 (0.81–0.96)	1.03 (0.98–1.07)	1.12 (1.04–1.20)
Hispanic	1.00 (0.92–1.09)	1.06 (1.01–1.11)	1.12 (1.04–1.20)
Other	0.73 (0.65–0.83)	0.88 (0.82–0.94)	0.93 (0.84–1.03)
	Non-persistence N = 17,589	Low adherence, overall population N = 17,589	Low adherence, among those with persistence N = 13,625
	2017–2018		
Non-Hispanic Asian	1 (ref)	1 (ref)	1 (ref)
Non-Hispanic White	0.74 (0.64–0.85)	0.78 (0.72–0.84)	0.75 (0.65–0.86)
Non-Hispanic Black	0.80 (0.68–0.94)	1.04 (0.95–1.14)	1.22 (1.05–1.42)
Hispanic	1.04 (0.88–1.23)	1.06 (0.96–1.17)	1.11 (0.94–1.31)
Other	0.68 (0.54–0.85)	0.84 (0.74–0.95)	0.91 (0.74–1.11)

Data in the table are risk ratios (95% CI) adjusted for calendar period of initiation, age, sex, antihypertensive medication class initiated during the follow-up period, antihypertensive medication regimen initiated during the follow-up period (single class, multiple classes with multiple pills, and fixed-dosed combination therapy), initiated with a 90-day fill, copay-per-day of supply, prevalent conditions, newly documented conditions, and Medicare Part D coverage.

### Sensitivity analyses

When defining non-persistence as not having medication available to take for ≥ 90 consecutive days during the follow-up period, the proportion of non-Hispanic Asian beneficiaries with non-persistence and non-Hispanic White beneficiaries with non-persistence decreased and increased, respectively, from 2011–2012 to 2017–2018 **(S5 Table).** After defining non-persistence as not having medication available to take during the last 60 days of the follow-up period, there was no evidence of a trend in non-persistence from 2011–2012 to 2017–2018 among non-Hispanic Asian beneficiaries whereas it increased among non-Hispanic White beneficiaries. When defining non-persistence as not having medication available to take for ≥60 consecutive days during follow-up, there was no evidence of a trend in non-persistence from 2011–2012 to 2017–2018 among non-Hispanic Asian beneficiaries or non-Hispanic White beneficiaries. For each alternative definition, non-Hispanic White beneficiaries were less likely than non-Hispanic Asian beneficiaries to have non-persistence to antihypertensive medication **(S6 Table).**

Median PDC increased from 2011–2012 to 2017–2018 among non-Hispanic Asian beneficiaries and non-Hispanic White beneficiaries **(S7 Table).** Compared with non-Hispanic Asian beneficiaries, non-Hispanic White beneficiaries were less likely to have low adherence in 2011–2018 and 2017–2018 **(S8 Table).**

### Characteristics associated with non-persistence and low adherence among non-Hispanic Asian Medicare beneficiaries

Among non-Hispanic Asian beneficiaries, treatment with multiple classes with multiple pills, initiating treatment with a 90-day fill, and Medicare Part D coverage gap were each associated with a lower likelihood of non-persistence (**S9 Table**). Low adherence was less common among non-Hispanic Asian beneficiaries whose initiation regimen included multiple classes with multiple pills, initiating treatment with a 90-day fill, and Medicare Part D coverage gap, whereas low adherence was more common for those whose antihypertensive medication initiation regimen included a loop diuretic, those with serious fall injury and with newly documented CKD (**S10 Table**). The characteristics associated with low adherence among those who were persistent are shown in **S11 Table**.

## Discussion

The current study has several findings with clinical and public health implications. First, the proportions of non-Hispanic Asian beneficiaries in the current study with non-persistence and low adherence in the year following initiating antihypertensive medication were high. Second, among non-Hispanic Asian Medicare beneficiaries, there was no evidence of a trend in non-persistence from 2011–2012 to 2017–2018. Third, in 2017–2018, Non-Hispanic Asian Medicare beneficiaries were more likely than non-Hispanic White and non-Hispanic Black beneficiaries, and beneficiaries with Other race/ethnicity to have non-persistence. Also, they were more likely than non-Hispanic White and beneficiaries with Other race/ethnicity to have low adherence. Fourth, several factors were associated with a lower risk of non-persistence and low adherence among non-Hispanic Asian beneficiaries including initiating an antihypertensive medication regimen with multiple classes and with a 90-day prescription, and a Medicare Part D coverage gap. Further, among non-Hispanic Asian beneficiaries, low adherence was more common for those taking a loop diuretic and those with newly documented CKD. This study demonstrates that non-persistence and low adherence are common among non-Hispanic Asian adults.

In NHANES 2017–2020, non-Hispanic Asian US adults were more likely to have uncontrolled BP when compared to non-Hispanic White US adults [2]. Antihypertensive medication non-persistence and low adherence are individual-level factors contributing to uncontrolled BP among adults diagnosed with hypertension [26]. However, there are scarce data on the proportion and temporal change in non-persistence and low adherence to antihypertensive medication among non-Hispanic Asian adults relative to other race/ethnicity groups. In the current study, there was no evidence of a change in antihypertensive medication persistence from 2011–2012 to 2017–2018 among non-Hispanic Asian Medicare beneficiaries. Although there was an improvement in adherence during this period, non-Hispanic Asian beneficiaries were more likely to have both non-persistence and low adherence compared to non-Hispanic White beneficiaries in 2017–2018. These findings suggest that non-persistence and low adherence remain common among non-Hispanic Asian adults, which may contribute to their worse BP control compared to non-Hispanic Whites.

We identified several modifiable factors that may be useful in clinical practice for increasing persistence and adherence. Initiating treatment with a 90-day fill may improve persistence and adherence through lowering the barrier to maintaining medication supply [27]. Avoiding the initial treatment with a loop diuretic, which often requires twice-daily dosing, may increase adherence [28, 29]. Also, initiating antihypertensive medication with a loop diuretic is not consistent with the 2017 American College of Cardiology/American Heart Association BP guideline which recommends ACE inhibitors or ARBs, calcium channel blockers, or thiazide diuretics as first-line antihypertensive agents [30]. Initiating antihypertensive medication with two or more classes, either as fixed-dose combination or with multiple pills, has been shown to more quickly achieve goal BP levels [31, 32]. In the current study, initiating an antihypertensive medication regimen with multiple classes using multiple pills was associated with better persistence and adherence among non-Hispanic Asian Medicare beneficiaries. Among non-Hispanic Asian adults, the relative risks for non-persistence and low adherence associated with initiating an antihypertensive medication regimen with multiple classes using multiple pills and fixed-dose combination pills were similar. These findings suggest that fixed-dose combination therapy may reduce both non-persistence and low adherence. Finally, the Medicare Part D coverage gap, also known as the donut hole, indicates a period where people are not being reimbursed for their medication. In the current study, patients who had a Medicare Part D coverage gap were less likely to have non-persistence or low adherence, indicating these patients were taking their antihypertensive medications. However, drug costs are an issue for many patients with hypertension [33, 34]. Eliminating the Part D coverage gap and providing continuous pharmacy coverage to patients with hypertension may result in improved persistence and medication adherence.

Increasing antihypertensive medication persistence and adherence requires addressing barriers at several levels: individual, organizational, community, and policy, which comprise the socio-ecological framework [35]. Individual barriers not only include behavioral factors (i.e. not taking antihypertensive medications as prescribed) but also cognitive/affective factors (i.e. lack of knowledge or not understanding the benefits and potential harms of antihypertensive medication and BP control, depression, cognitive impairment, low health literacy, etc.) and provider-level factors (treatment inertia, lack of knowledge about BP control, uncertainty regarding treatment goals and guideline recommendations, inadequate time, etc.). Therefore, improving BP control requires a multi-faceted approach, and improving persistence and adherence to antihypertensive medication among older non-Hispanic Asian adults may only represent one area that needs to be addressed. Finally, interventions that improve persistence and adherence among non-Hispanic Asian adults most likely have to be culturally appropriate and tailored to this specific race/ethnic group [11].

There are several strengths associated with the current analysis. Medicare data provide a high level of generalizability to older US adults [36]. The longitudinal data for Medicare beneficiaries allowed us to identify patients who were initiating antihypertensive medication and follow them over time. Medicare has a large number of non-Hispanic Asian beneficiaries, which provided sufficient statistical power for this analysis. Despite these strengths, the results for the current study should be interpreted in the context of potential limitations. Race/ethnicity was not determined by self-report, which has been recommended in a data brief by the US Department of Health and Human Services Office of Inspector General [37]. Non-persistence and low adherence were determined only by filled prescriptions for antihypertensive medication. We were unable to confirm whether beneficiaries actually took their medication or if antihypertensive medication was prescribed but not filled [38]. BP levels and physician notes were not available. Some patients may have discontinued medication based on their BP levels and physician’s advice. Additionally, non-Hispanic Asian adults represent a heterogeneous group comprised of over 40 ethnic sub-groups based on, indigeneity, ancestry, acculturation and other factors [11]. Medicare does not have information on these factors or if beneficiaries spoke English or medication beliefs. Qualitative research suggests beliefs about complementary and alternative medication may influence medication adherence among Asian-American adults [39]. Data were not available for younger adults and the results of the current study apply only to older US adults. Medicare only has claims for prescriptions filled and does not have data on medications prescribed. Therefore, we could not study primary non-adherence to antihypertensive medication. It is possible that the analyses overestimated persistence and adherence for those who initiated two or more antihypertension medication classes since the definitions of persistence and adherence were dependent on having any antihypertensive medication available to take during the follow-up period, regardless of the number of antihypertensive medications initiated. Finally, we were not able to determine the underlying reasons for non-Hispanic Asian beneficiaries being more likely to have non-persistence and low adherence than non-Hispanic White beneficiaries. Future studies may be helpful to identify social determinations of health, cognitive/affective and behavioral factors, and clinician- or health system-related factors to these racial differences.

In conclusion, a substantial percentage of non-Hispanic Asian beneficiaries had non-persistence and low adherence to antihypertensive medication in the year following treatment initiation. Non-Hispanic Asian beneficiaries were more likely to have non-persistence and low adherence to antihypertensive medication when compared with non-Hispanic White beneficiaries. Clinicians should consider prescribing antihypertensive medication with a 90-day fill, use of multiple drug classes in an initial antihypertensive medication regimen, and not using loop diuretics to increase persistence and adherence among older non-Hispanic Asian adults.

## Supporting information

S1 File Statistical code. (ZIP)

S1 Table Flowchart of Medicare beneficiaries included in the current analysis by calendar year and race/ethnicity. (PDF)

S2 Table Sample sizes used for estimating the race/ethnicity-specific proportion of beneficiaries with non-persistence, low adherence who initiated antihypertensive medication, and low adherence among those who were persistent by two-year calendar periods. (PDF)

S3 Table Race/ethnicity-specific proportion of women and men with non-persistence and low adherence who initiated antihypertensive medication and low adherence among those who were persistent by two-year calendar periods. (PDF)

S4 Table Multivariable-adjusted risk ratios (95% CI) for non-persistence and low adherence among women and men who initiated antihypertensive medication and low adherence among women and men with persistence in 2011–2018 and 2017–2018. (PDF)

S5 Table Race/ethnicity-specific proportion of beneficiaries with non-persistence and low adherence among those who were persistent by two-year calendar periods, using sensitivity analysis definitions of non-persistence. (PDF)

S6 Table Multivariable-adjusted risk ratios (95% CI) for non-persistence and low adherence among beneficiaries with persistence in 2011–2018 and 2017–2018, using sensitivity analysis definitions of non-persistence. (PDF)

S7 Table Race/ethnicity-specific median (25th, 75th percentiles) proportion of days covered among beneficiaries that initiated antihypertensive medication and those who were persistent by two-year calendar periods. (PDF)

S8 Table Multivariable-adjusted difference in median (95% confidence interval) proportion of days covered among beneficiaries who initiated antihypertensive medication and among beneficiaries with persistence in 2011–2018 and 2017–2018. (PDF)

S9 Table Adjusted risk ratios for non-persistence associated with beneficiary characteristics within race/ethnicity groups. (PDF)

S10 Table Race/ethnicity-specific adjusted risk ratios for having low adherence among all beneficiaries initiating antihypertensive medication. (PDF)

S11 Table Race/ethnicity-specific adjusted risk ratios for low adherence among beneficiaries who were persistent to their antihypertensive medication. (PDF)
