# Supplementary material for: Antihypertensive medication persistence and adherence among non-Hispanic Asian US patients with hypertension and fee-for-service Medicare health insurance
Source: PLoS One. 2024 Mar 20;19(3):e0300372. doi: 10.1371/journal.pone.0300372 (PMC10954118; doi:10.1371/journal.pone.0300372)
Supplement: S1 Table — (PDF) [file pone.0300372.s002.pdf]

**S1 Table. Flowchart of Medicare beneficiaries included in the current analysis by calendar year and race/ethnicity.**

| | Calendar year of antihypertensive medication initiation | | | | | | | |
|---|---|---|---|---|---|---|---|---|
| | 2011 | 2012 | 2013 | 2014 | 2015 | 2016 | 2017 | 2018 |
| **Non-Hispanic Asian** | | | | | | | | |
| Filled antihypertensive medication | 35,824 | 38,374 | 41,620 | 44,335 | 44,866 | 49,664 | 52,608 | 55,777 |
| No data anomalies* | 35,604 | 38,144 | 41,388 | 44,103 | 44,646 | 49,453 | 52,400 | 55,585 |
| Age ≥ 66 | 31,308 | 33,071 | 35,733 | 38,154 | 38,423 | 42,977 | 45,724 | 48,615 |
| Had Medicare Part A, B, D and did not have C coverage | | | | | | | | |
| For the 365-day look-back period | 14,820 | 15,264 | 15,789 | 16,723 | 14,899 | 15,830 | 17,809 | 18,739 |
| For the 365-day follow-up period | 13,589 | 13,735 | 14,206 | 13,066 | 13,024 | 14,204 | 16,096 | 16,232 |
| Residence in the US | 13,523 | 13,677 | 14,152 | 13,016 | 12,976 | 14,141 | 16,025 | 16,167 |
| Did not die before the end of the follow-up period | 13,496 | 13,649 | 14,137 | 12,982 | 12,954 | 14,113 | 15,991 | 16,139 |
| History of hypertension | 9,942 | 10,319 | 10,566 | 9,611 | 9,406 | 10,306 | 11,218 | 11,718 |
| Not taking antihypertensive medication in the look-back period | 292 | 337 | 321 | 268 | 272 | 312 | 333 | 375 |
| Keep only the first record for each beneficiary | 292 | 331 | 296 | 246 | 227 | 270 | 280 | 318 |
| **Non-Hispanic White** | | | | | | | | |
| Filled antihypertensive medication | 874,064 | 928,336 | 1,042,532 | 1,091,259 | 1,045,833 | 1,161,986 | 1,195,550 | 1,227,889 |
| No data anomalies* | 870,941 | 925,189 | 1,039,273 | 1,088,097 | 1,043,163 | 1,159,124 | 1,192,854 | 1,225,401 |
| Age ≥ 66 | 720,868 | 757,717 | 857,515 | 902,561 | 856,339 | 963,697 | 992,694 | 1,023,058 |

| | | | | | | | | |
|---|---|---|---|---|---|---|---|---|
| Medicare Part A, B, D not C coverage | | | | | | | | |
| For the 365-day look-back period | 390,883 | 405,816 | 424,961 | 486,682 | 455,772 | 479,173 | 530,576 | 535,282 |
| For the 365-day follow-up period | 353,842 | 367,189 | 385,252 | 397,843 | 415,787 | 431,101 | 478,661 | 452,651 |
| Residence in the US | 353,760 | 367,115 | 385,162 | 397,759 | 415,692 | 430,997 | 478,550 | 452,561 |
| Did not die before the end of the follow-up period | 352,746 | 365,903 | 384,127 | 396,621 | 414,654 | 429,974 | 477,249 | 451,535 |
| History of hypertension | 226,507 | 235,602 | 248,437 | 256,228 | 266,622 | 278,841 | 301,231 | 303,762 |
| Not taking antihypertensive meds in look-back period | 7,500 | 7,575 | 7,848 | 6,982 | 7,237 | 7,661 | 8,064 | 8,187 |
| Keep only the first record for each beneficiary | 7,500 | 7,403 | 7,334 | 6,393 | 6,497 | 6,743 | 7,045 | 7,085 |
| **Non-Hispanic Black** | | | | | | | | |
| Filled antihypertensive medication | 137,939 | 147,272 | 164,561 | 173,194 | 170,218 | 187,184 | 193,239 | 199,453 |
| No data anomalies* | 136,789 | 146,093 | 163,290 | 171,915 | 169,055 | 185,954 | 192,057 | 198,289 |
| Age $\geq$ 66 | 89,367 | 94,666 | 106,996 | 112,978 | 109,029 | 123,902 | 129,650 | 135,476 |
| Medicare Part A, B, D not C coverage | | | | | | | | |
| For the 365-day look-back period | 40,377 | 41,249 | 42,734 | 48,575 | 44,218 | 45,637 | 49,210 | 47,966 |
| For the 365-day follow-up period | 35,086 | 35,625 | 36,602 | 36,945 | 38,043 | 38,425 | 41,189 | 37,816 |
| Residence in the US | 34,987 | 35,516 | 36,487 | 36,826 | 37,785 | 38,156 | 40,903 | 37,539 |
| Did not die before the end of the follow-up period | 34,871 | 35,401 | 36,384 | 36,710 | 37,702 | 38,041 | 40,778 | 37,456 |

| | | | | | | | | |
|---|---|---|---|---|---|---|---|---|
| History of hypertension | 27,330 | 28,382 | 29,075 | 29,069 | 29,624 | 30,113 | 31,117 | 29,963 |
| Not taking antihypertensive meds in look-back period | 783 | 820 | 801 | 811 | 744 | 782 | 799 | 804 |
| Keep only the first record for each beneficiary | 783 | 802 | 740 | 749 | 661 | 652 | 706 | 699 |
| **Hispanic** | | | | | | | | |
| Filled antihypertensive medication | 112,711 | 120,404 | 130,224 | 137,268 | 138,306 | 150,617 | 157,294 | 164,632 |
| No data anomalies* | 111,536 | 119,196 | 128,971 | 136,007 | 137,126 | 149,425 | 156,116 | 163,459 |
| Age ≥ 66 | 84,745 | 89,967 | 97,662 | 103,232 | 102,984 | 114,174 | 120,609 | 127,009 |
| Medicare Part A, B, D not C coverage | | | | | | | | |
| For the 365-day look-back period | 30,249 | 30,938 | 31,158 | 32,342 | 28,054 | 28,865 | 31,927 | 32,013 |
| For the 365-day follow-up period | 26,767 | 26,872 | 26,979 | 24,051 | 23,535 | 25,211 | 27,397 | 26,164 |
| Residence in the US | 25,925 | 26,133 | 26,260 | 23,647 | 23,124 | 24,779 | 26,912 | 25,679 |
| Did not die before the end of the follow-up period | 25,874 | 26,060 | 26,191 | 23,595 | 23,072 | 24,719 | 26,846 | 25,619 |
| History of hypertension | 18,747 | 19,407 | 19,523 | 17,396 | 16,572 | 18,103 | 19,231 | 18,944 |
| Not taking antihypertensive meds in look-back period | 636 | 669 | 633 | 479 | 528 | 532 | 568 | 549 |
| Keep only the first record for each beneficiary | 636 | 649 | 589 | 437 | 454 | 471 | 508 | 468 |
| **Other** | | | | | | | | |
| Filled antihypertensive medication | 15,631 | 18,117 | 22,080 | 25,620 | 28,167 | 32,767 | 36,708 | 40,556 |
| No data anomalies* | 15,539 | 18,035 | 21,994 | 25,532 | 28,081 | 32,675 | 36,617 | 40,465 |

| | | | | | | | | |
|---|---|---|---|---|---|---|---|---|
| Age ≥ 66 | 10,888 | 12,083 | 14,606 | 17,770 | 20,261 | 24,593 | 28,507 | 32,427 |
| Medicare Part A, B, D not C coverage | | | | | | | | |
| 365-day look-back period | 5,362 | 5,774 | 6,645 | 8,494 | 9,535 | 11,366 | 13,866 | 15,704 |
| 365-day follow-up period | 4,848 | 5,249 | 6,055 | 7,224 | 8,729 | 10,459 | 12,781 | 14,094 |
| Residence in the US | 4,831 | 5,228 | 6,031 | 7,206 | 8,701 | 10,428 | 12,747 | 14,067 |
| Did not die before the end of the follow-up period | 4,825 | 5,217 | 6,017 | 7,189 | 8,689 | 10,415 | 12,727 | 14,051 |
| History of hypertension | 3,202 | 3,568 | 3,986 | 4,593 | 5,435 | 6,572 | 7,847 | 8,821 |
| Not taking antihypertensive meds in look-back period | 141 | 141 | 134 | 159 | 199 | 233 | 245 | 285 |
| Keep only the first record for each beneficiary | 141 | 135 | 126 | 150 | 177 | 214 | 225 | 255 |

*Data anomalies include having more than one birth date, more than one death date (for those who died), more than one sex or race/ethnicity.

Data are expressed as number of patients in each race/ethnic group in each calendar year who met the eligibility criteria.
