# Supplementary material for: Antihypertensive medication persistence and adherence among non-Hispanic Asian US patients with hypertension and fee-for-service Medicare health insurance
Source: PLoS One. 2024 Mar 20;19(3):e0300372. doi: 10.1371/journal.pone.0300372 (PMC10954118; doi:10.1371/journal.pone.0300372)
Supplement: S2 Table — (PDF) [file pone.0300372.s003.pdf]

S2 Table. Sample sizes used for estimating the race/ethnicity-specific proportion of beneficiaries with non-persistence, low adherence who initiated antihypertensive medication, and low adherence among those who were persistent by two-year calendar periods.

	Race/ethnicity				
	Non-Hispanic Asian	Non-Hispanic White	Non-Hispanic Black	Hispanic	Other
Non-persistence, n					
2011-2012	623	14,903	1,585	1,285	276
2013-2014	583	14,693	1,596	1,101	291
2015-2016	575	14,737	1,500	1,053	428
2017-2018	692	16,065	1,587	1,105	527
Low adherence among all beneficiaries who initiated antihypertensive medication, n					
2011-2012	623	14,903	1,585	1,285	276
2013-2014	583	14,693	1,596	1,101	291
2015-2016	575	14,737	1,500	1,053	428
2017-2018	692	16,065	1,587	1,105	527
Low adherence among those who had persistence, n					
2011-2012	442	11,754	1,184	920	214
2013-2014	434	11,556	1,226	793	232
2015-2016	429	11,440	1,087	783	347
2017-2018	507	12,397	1,212	785	421
