# Supplementary material for: Antihypertensive medication persistence and adherence among non-Hispanic Asian US patients with hypertension and fee-for-service Medicare health insurance
Source: PLoS One. 2024 Mar 20;19(3):e0300372. doi: 10.1371/journal.pone.0300372 (PMC10954118; doi:10.1371/journal.pone.0300372)
Supplement: S3 Table — (PDF) [file pone.0300372.s004.pdf]

S3 Table. Race/ethnicity-specific proportion of women and men with non-persistence and low adherence who initiated antihypertensive medication and low adherence among those who were persistent by two-year calendar periods.

	Race/ethnicity				
	Non-Hispanic Asian	Non-Hispanic White	Non-Hispanic Black	Hispanic	Other
Non-persistence, n (%)					
**Women**					
2011-2012	111 (28.8%)	1,929 (20.6%)***	235 (23.6%)*	215 (27.3%)	34 (20.7%)
2013-2014	76 (23.9%)	1,806 (20.6%)	216 (22.2%)	167 (25.3%)	34 (21.0%)
2015-2016	71 (22.1%)	1,837 (21.7%)	235 (27.5%)	153 (25.7%)	34 (17.0%)
2017-2018	113 (28.5%)	2,059 (22.4%)**	212 (22.8%)*	176 (26.7%)	50 (19.6%)*
P-trend	0.843	0.001	0.635	0.790	0.617
**Men**					
2011-2012	70 (29.5%)	1,220 (22.0%)**	166 (28.2%)	150 (30.1%)	28 (25.0%)
2013-2014	73 (27.5%)	1,331 (22.5%)	154 (24.7%)	141 (32.0%)	25 (19.4%)
2015-2016	75 (29.5%)	1,460 (23.3%)*	178 (27.6%)	117 (25.6%)	47 (20.6%)*
2017-2018	72 (24.3%)	1,609 (23.4%)	163 (24.7%)	144 (32.4%)*	56 (20.6%)
P-trend	0.245	0.036	0.339	0.984	0.501
Low adherence among all beneficiaries who initiated antihypertensive medication, n (%)					
**Women**					
2011-2012	231 (59.8%)	4,250 (45.4%)***	578 (58.0%)	481 (61.1%)	83 (50.6%)*
2013-2014	166 (52.2%)	3,945 (45.0%)*	535 (55.0%)	365 (55.3%)	79 (48.8%)
2015-2016	164 (51.1%)	3,769 (44.5%)*	499 (58.4%)*	337 (56.5%)	109 (54.5%)
2017-2018	213 (53.8%)	4,015 (43.7%)***	517 (55.7%)	368 (55.8%)	111 (43.5%)*
P-trend	0.090	0.013	0.620	0.058	0.229
**Men**					
2011-2012	131 (55.3%)	2,592 (46.7%)*	356 (60.5%)	323 (64.9%)*	60 (53.6%)
2013-2014	150 (56.6%)	2,689 (45.4%)***	348 (55.9%)	285 (64.6%)*	70 (54.3%)
2015-2016	143 (56.3%)	2,919 (46.5%)**	389 (60.2%)	269 (58.9%)	92 (40.4%)***

2017-2018	144 (48.6%)	3,088 (45.0%)	389 (59.0%)**	269 (60.4%)**	132 (48.5%)
P-trend	0.112	0.132	0.986	0.058	0.182
Low adherence among those who had persistence, n (%)					
**Women**					
2011-2012	120 (43.6%)	2,322 (31.3%)***	343 (45.0%)	266 (46.5%)	49 (37.7%)
2013-2014	90 (37.2%)	2,139 (30.7%)*	319 (42.1%)	198 (40.2%)	45 (35.2%)
2015-2016	93 (37.2%)	1,932 (29.2%)**	264 (42.6%)	184 (41.5%)	75 (45.2%)
2017-2018	100 (35.3%)	1,956 (27.4%)**	305 (42.6%)*	192 (39.7%)	61 (29.8%)
P-trend	0.055	<0.001	0.405	0.040	0.268
**Men**					
2011-2012	61 (36.5%)	1,372 (31.7%)	190 (45.0%)	173 (49.7%)**	32 (38.1%)
2013-2014	77 (40.1%)	1,358 (29.5%)**	194 (41.4%)	144 (48.0%)	45 (43.3%)
2015-2016	68 (38.0%)	1,459 (30.3%)*	211 (45.1%)	152 (44.7%)	45 (24.9%)**
2017-2018	72 (32.1%)	1,479 (28.1%)	226 (45.6%)**	125 (41.5%)*	76 (35.2%)
P-trend	0.260	0.001	0.568	0.026	0.245

Data are expressed as number (percent) for outcomes. P-trend represents the trend across the calendar periods.
* p-value of 0.01 to 0.05 compared to non-Hispanic Asians within each calendar period;
** p-value of 0.001 to 0.01 compared to non-Hispanic Asians within each calendar period;
*** p-value <0.001 compared to non-Hispanic Asians within each calendar period.
