# Supplementary material for: Antihypertensive medication persistence and adherence among non-Hispanic Asian US patients with hypertension and fee-for-service Medicare health insurance
Source: PLoS One. 2024 Mar 20;19(3):e0300372. doi: 10.1371/journal.pone.0300372 (PMC10954118; doi:10.1371/journal.pone.0300372)
Supplement: S4 Table — (PDF) [file pone.0300372.s005.pdf]

S4 Table. Multivariable-adjusted risk ratios (95% CI) for non-persistence and low adherence among women and men who initiated antihypertensive medication and low adherence among women and men with persistence in 2011-2018 and 2017-2018.

	Adjusted risk ratio (95% confidence interval)		
	Non-persistence	Low adherence, overall population	Low adherence, among those with persistence
	2011-2018		
**Women**			
Non-Hispanic Asian	1 (ref)	1 (ref)	1 (ref)
Non-Hispanic White	0.73 (0.67-0.80)	0.77 (0.73-0.81)	0.72 (0.67-0.79)
Non-Hispanic Black	0.86 (0.78-0.96)	1.00 (0.94-1.06)	1.07 (0.97-1.17)
Hispanic	0.96 (0.86-1.07)	1.01 (0.95-1.07)	1.04 (0.94-1.14)
Other	0.71 (0.60-0.85)	0.87 (0.80-0.96)	0.93 (0.81-1.06)
**Men**			
Non-Hispanic Asian	1 (ref)	1 (ref)	1 (ref)
Non-Hispanic White	0.76 (0.68-0.85)	0.80 (0.75-0.85)	0.78 (0.70-0.86)
Non-Hispanic Black	0.90 (0.79-1.02)	1.06 (0.99-1.14)	1.20 (1.07-1.35)
Hispanic	1.06 (0.93-1.21)	1.14 (1.06-1.22)	1.25 (1.11-1.40)
Other	0.76 (0.63-0.90)	0.89 (0.81-0.98)	0.95 (0.81-1.11)
	2017-2018		
**Women**			
Non-Hispanic Asian	1 (ref)	1 (ref)	1 (ref)
Non-Hispanic White	0.67 (0.56-0.80)	0.74 (0.66-0.82)	0.72 (0.60-0.86)
Non-Hispanic Black	0.71 (0.58-0.88)	0.96 (0.85-1.08)	1.11 (0.91-1.34)
Hispanic	0.89 (0.72-1.11)	0.98 (0.87-1.11)	1.05 (0.85-1.29)
Other	0.59 (0.43-0.82)	0.74 (0.61-0.88)	0.77 (0.58-1.02)
**Men**			
Non-Hispanic Asian	1 (ref)	1 (ref)	1 (ref)
Non-Hispanic White	0.85 (0.68-1.08)	0.85 (0.74-0.97)	0.80 (0.65-1.00)
Non-Hispanic Black	0.94 (0.72-1.23)	1.17 (1.01-1.35)	1.39 (1.10-1.77)
Hispanic	1.29 (0.99-1.69)	1.19 (1.02-1.39)	1.20 (0.93-1.56)

Other	0.82 (0.59-1.14)	0.99 (0.82-1.19)	1.10 (0.82-1.47)

Data in the table are risk ratios (95% CI) adjusted for calendar period of initiation, age, sex, antihypertensive medication class initiated during the follow-up period, antihypertensive medication regimen initiated during the follow-up period (single class, multiple classes with multiple pills, and fixed-dosed combination therapy), initiated with a 90-day fill, copay-per-day of supply, prevalent conditions, newly documented conditions, and Medicare Part D coverage gap.
