# Supplementary material for: Antihypertensive medication persistence and adherence among non-Hispanic Asian US patients with hypertension and fee-for-service Medicare health insurance
Source: PLoS One. 2024 Mar 20;19(3):e0300372. doi: 10.1371/journal.pone.0300372 (PMC10954118; doi:10.1371/journal.pone.0300372)
Supplement: S5 Table — (PDF) [file pone.0300372.s006.pdf]

S5 Table. Race/ethnicity-specific proportion of beneficiaries with non-persistence and low adherence among those who were persistent by two-year calendar periods, using sensitivity analysis definitions of non-persistence.

	Race/ethnicity				
	Non-Hispanic Asian	Non-Hispanic White	Non-Hispanic Black	Hispanic	Other
**Sensitivity analysis definition 1 of non-persistence: not having medication available to take for ≥90 consecutive days at any time during follow-up.**					
Non-persistence, n (%)					
2011-2012	284 (45.6%)	5,092 (34.2%)***	682 (43.0%)	614 (47.8%)	99 (35.9%)**
2013-2014	245 (42.0%)	5,020 (34.2%)***	629 (39.4%)	486 (44.1%)	107 (36.8%)
2015-2016	230 (40.0%)	5,137 (34.9%)*	675 (45.0%)*	432 (41.0%)	142 (33.2%)*
2017-2018	269 (38.9%)	5,642 (35.1%)*	682 (43.0%)	510 (46.2%)**	179 (34.0%)
P-trend	0.011	0.039	0.338	0.183	0.419
Low adherence among those who had persistence, n (%)					
2011-2012	78 (23.0%)	1,752 (17.9%)*	252 (27.9%)	190 (28.3%)	44 (24.9%)
2013-2014	71 (21.0%)	1,614 (16.7%)*	254 (26.3%)	164 (26.7%)	42 (22.8%)
2015-2016	77 (22.3%)	1,551 (16.2%)**	213 (25.8%)	174 (28.0%)	59 (20.6%)
2017-2018	88 (20.8%)	1,461 (14.0%)***	224 (24.8%)	127 (21.3%)	64 (18.4%)
P-trend	0.570	<.0001	0.130	0.014	0.065
**Sensitivity analysis definition 2 of non-persistence: not having medication available to take during the last 60 days of the follow-up period.**					
Non-persistence, n (%)					
2011-2012	198 (31.8%)	3,521 (23.6%)***	448 (28.3%)	395 (30.7%)	66 (23.9%)*
2013-2014	167 (28.6%)	3,507 (23.9%)**	419 (26.3%)	341 (31.0%)	71 (24.4%)
2015-2016	164 (28.5%)	3,661 (24.8%)*	468 (31.2%)	311 (29.5%)	90 (21.0%)**
2017-2018	201 (29.0%)	4,022 (25.0%)*	428 (27.0%)	356 (32.2%)	118 (22.4%)**
P-trend	0.310	0.001	0.858	0.626	0.459
Low adherence among those who had persistence, n (%)					
2011-2012	166 (39.1%)	3,359 (29.5%)***	486 (42.7%)	410 (46.1%)*	77 (36.7%)

2013-2014	150 (36.1%)	3,167 (28.3%)***	464 (39.4%)	311 (40.9%)	79 (35.9%)
2015-2016	144 (35.0%)	3,064 (27.7%)**	425 (41.2%)*	297 (40.0%)	111 (32.8%)
2017-2018	157 (32.0%)	3,121 (25.9%)**	482 (41.6%)***	282 (37.7%)*	126 (30.8%)
P-trend	0.026	<.0001	0.804	0.001	0.093
**Sensitivity analysis definition 3 of non-persistence: not having medication available to take for ≥60 consecutive days at any time during follow-up.**					
Non-persistence, n (%)					
2011-2012	330 (53.0%)	6,222 (41.7%)***	825 (52.1%)	720 (56.0%)	117 (42.4%)**
2013-2014	291 (49.9%)	6,065 (41.3%)***	785 (49.2%)	591 (53.7%)	136 (46.7%)
2015-2016	280 (48.7%)	6,156 (41.8%)***	810 (54.0%)*	548 (52.0%)	177 (41.4%)*
2017-2018	335 (48.4%)	6,696 (41.7%)***	832 (52.4%)	592 (53.6%)*	217 (41.2%)*
P-trend	0.094	0.873	0.297	0.151	0.381
Low adherence among those who had persistence, n (%)					
2011-2012	37 (12.6%)	786 (9.1%)*	119 (15.7%)	92 (16.3%)	28 (17.6%)
2013-2014	34 (11.6%)	716 (8.3%)*	112 (13.8%)	73 (14.3%)	14 (9.0%)
2015-2016	31 (10.5%)	669 (7.8%)	96 (13.9%)	69 (13.7%)	28 (11.2%)
2017-2018	28 (7.8%)	581 (6.2%)	92 (12.2%)*	50 (9.7%)	28 (9.0%)
P-trend	0.040	<.0001	0.066	0.002	0.021

Data are expressed as number (percent) for outcomes. P-trend represents the trend across the calendar periods.
Low adherence to antihypertensive medication was defined by an interval-based proportion of days covered (PDC) <80%.
* p-value of 0.01 to 0.05 compared to non-Hispanic Asians within each calendar period;
** p-value of 0.001 to < 0.01 compared to non-Hispanic Asians within each calendar period;
*** p-value <0.001 compared to non-Hispanic Asians within each calendar period.
