# Supplementary material for: Antihypertensive medication persistence and adherence among non-Hispanic Asian US patients with hypertension and fee-for-service Medicare health insurance
Source: PLoS One. 2024 Mar 20;19(3):e0300372. doi: 10.1371/journal.pone.0300372 (PMC10954118; doi:10.1371/journal.pone.0300372)
Supplement: S6 Table — (PDF) [file pone.0300372.s007.pdf]

S6 Table. Multivariable-adjusted risk ratios (95% CI) for non-persistence and low adherence among beneficiaries with persistence in 2011-2018 and 2017-2018, using sensitivity analysis definitions of non-persistence.

	Adjusted risk ratio (95% confidence interval)	
**Sensitivity analysis definition 1 of non-persistence: not having medication available to take for ≥90 consecutive days at any time during follow-up.**		
	Non-persistence N=69,687	Low adherence, among those with persistence N=45,092
	2011-2018	
Non-Hispanic Asian	1 (ref)	1 (ref)
Non-Hispanic White	0.76 (0.73-0.80)	0.72 (0.64-0.80)
Non-Hispanic Black	0.97 (0.92-1.03)	1.18 (1.04-1.32)
Hispanic	1.05 (0.99-1.11)	1.14 (1.01-1.30)
Other	0.81 (0.74-0.88)	0.97 (0.83-1.15)
	Non-persistence N=17,589	Low adherence, among those with persistence N=11,434
	2017-2018	
Non-Hispanic Asian	1 (ref)	1 (ref)
Non-Hispanic White	0.80 (0.72-0.89)	0.63 (0.51-0.77)
Non-Hispanic Black	1.02 (0.90-1.15)	1.14 (0.90-1.45)
Hispanic	1.14 (1.01-1.29)	0.91 (0.69-1.18)
Other	0.80 (0.68-0.94)	0.85 (0.62-1.17)
**Sensitivity analysis definition 2 of non-persistence: not having medication available to take during the last 60 days of the follow-up period.**		
	Non-persistence N=69,687	Low adherence, among those with persistence N=52,473
	2011-2018	
Non-Hispanic Asian	1 (ref)	1 (ref)
Non-Hispanic White	0.76 (0.71-0.81)	0.74 (0.69-0.79)
Non-Hispanic Black	0.91 (0.84-0.98)	1.12 (1.04-1.21)
Hispanic	1.01 (0.93-1.09)	1.12 (1.03-1.21)
Other	0.75 (0.67-0.84)	0.93 (0.83-1.04)

	Non-persistence<br>N=17,589	Low adherence, among those with persistence<br>N=17,589
	2017-2018	
Non-Hispanic Asian	1 (ref)	1 (ref)
Non-Hispanic White	0.76 (0.67-0.87)	0.73 (0.63-0.84)
Non-Hispanic Black	0.85 (0.73-0.99)	1.21 (1.03-1.42)
Hispanic	1.06 (0.90-1.24)	1.09 (0.91-1.29)
Other	0.71 (0.57-0.88)	0.90 (0.72-1.11)
**Sensitivity analysis definition 3 of non-persistence: not having medication available to take for $\geq$60 consecutive days at any time during follow-up.**		
	Non-persistence<br>N=69,687	Low adherence, among those with persistence<br>N=39,970
	2011-2018	
Non-Hispanic Asian	1 (ref)	1 (ref)
Non-Hispanic White	0.78 (0.75-0.81)	0.70 (0.59-0.84)
Non-Hispanic Black	1.00 (0.95-1.05)	1.24 (1.02-1.50)
Hispanic	1.05 (1.00-1.10)	1.19 (0.97-1.45)
Other	0.84 (0.78-0.90)	1.06 (0.82-1.38)
	Non-persistence<br>N=17,589	Low adherence, among those with persistence<br>N=10,237
	2017-2018	
Non-Hispanic Asian	1 (ref)	1 (ref)
Non-Hispanic White	0.78 (0.71-0.85)	0.73 (0.49-1.10)
Non-Hispanic Black	1.01 (0.91-1.11)	1.48 (0.95-2.30)
Hispanic	1.05 (0.94-1.16)	1.15 (0.71-1.88)
Other	0.79 (0.69-0.91)	1.16 (0.67-1.98)

Data in the table are risk ratios (95% CI) adjusted for calendar period of initiation (for 2011-2018 analysis), age, sex, antihypertensive medication class initiated during the follow-up period, antihypertensive medication regimen initiated during the follow-up period (single class, multiple classes with multiple pills, and fixed-dosed combination therapy), initiated with a 90-day fill, copay-per-day of supply, prevalent conditions, newly documented conditions, and Medicare Part D coverage. Low adherence to antihypertensive medication was defined by an interval-based proportion of days covered (PDC) <80%.
