# Supplementary material for: Antihypertensive medication persistence and adherence among non-Hispanic Asian US patients with hypertension and fee-for-service Medicare health insurance
Source: PLoS One. 2024 Mar 20;19(3):e0300372. doi: 10.1371/journal.pone.0300372 (PMC10954118; doi:10.1371/journal.pone.0300372)
Supplement: S7 Table — (PDF) [file pone.0300372.s008.pdf]

S7 Table. Race/ethnicity-specific median (25th, 75th percentiles) proportion of days covered among beneficiaries that initiated antihypertensive medication and those who were persistent by two-year calendar periods.

	Race/ethnicity				
	Non-Hispanic Asian	Non-Hispanic White	Non-Hispanic Black	Hispanic	Other
Median (25th, 75th percentile) PDC among all beneficiaries who initiated antihypertensive medication					
2011-2012	0.71 (0.32, 0.94)	0.84 (0.49, 0.98)***	0.71 (0.33, 0.93)	0.66 (0.30, 0.90)	0.78 (0.46, 0.97)
2013-2014	0.75 (0.41, 0.97)	0.85 (0.49, 0.98)**	0.74 (0.39, 0.95)	0.71 (0.37, 0.93)	0.78 (0.45, 0.96)
2015-2016	0.75 (0.44, 0.96)	0.85 (0.49, 0.98)**	0.68 (0.33, 0.93)*	0.73 (0.41, 0.95)	0.83 (0.49, 0.98)
2017-2018	0.78 (0.42, 0.98)	0.86 (0.49, 0.99)*	0.74 (0.41, 0.95)*	0.71 (0.36, 0.96)*	0.88 (0.49, 0.98)
P-trend	0.013	<0.001	0.162	0.001	0.004
Median (25th, 75th percentile) PDC among those who had persistence					
2011-2012	0.86 (0.65, 0.98)	0.92 (0.74, 0.99)***	0.84 (0.61, 0.96)	0.82 (0.59, 0.95)**	0.87 (0.71, 0.98)
2013-2014	0.89 (0.68, 0.98)	0.93 (0.74, 0.99)*	0.86 (0.63, 0.97)	0.84 (0.64, 0.97)*	0.88 (0.67, 0.98)
2015-2016	0.90 (0.70, 0.98)	0.93 (0.75, 0.99)***	0.83 (0.59, 0.97)**	0.84 (0.64, 0.98)	0.90 (0.74, 0.99)
2017-2018	0.91 (0.74, 0.99)	0.95 (0.77, 1.00)	0.84 (0.64, 0.98)***	0.89 (0.65, 0.98)**	0.94 (0.74, 0.99)
P-trend	0.016	<0.001	0.253	<0.001	<0.001

P-trend represents the trend across the calendar periods.
Non-persistence was defined as not having antihypertensive medication available to take in the last 90 days of the follow-up period.
* p-value of 0.01 to 0.05 compared to non-Hispanic Asians within each calendar period;
** p-value of 0.001 to 0.01 compared to non-Hispanic Asians within each calendar period;
*** p-value <0.001 compared to non-Hispanic Asians within each calendar period.
Abbreviations: PDC, interval-based proportion of days covered
