# Supplementary material for: Antihypertensive medication persistence and adherence among non-Hispanic Asian US patients with hypertension and fee-for-service Medicare health insurance
Source: PLoS One. 2024 Mar 20;19(3):e0300372. doi: 10.1371/journal.pone.0300372 (PMC10954118; doi:10.1371/journal.pone.0300372)
Supplement: S8 Table — (PDF) [file pone.0300372.s009.pdf]

S8 Table. Multivariable-adjusted difference in median (95% confidence interval) proportion of days covered among beneficiaries who initiated antihypertensive medication and among beneficiaries with persistence in 2011-2018 and 2017-2018.

	Adjusted difference in median PDC (95% confidence interval)	
	Overall population	Among those with persistence
	2011-2018	
Non-Hispanic Asian	0 (ref)	0 (ref)
Non-Hispanic White	0.12 (0.09, 0.14)	0.05 (0.03, 0.06)
Non-Hispanic Black	-0.01 (-0.04, 0.02)	-0.03 (-0.05, -0.02)
Hispanic	-0.04 (-0.07, -0.003)	-0.04 (-0.05, -0.02)
Other	0.08 (0.04, 0.11)	0.03 (0.01, 0.05)
	Overall population	Among those with persistence
	2017-2018	
Non-Hispanic Asian	0 (ref)	0 (ref)
Non-Hispanic White	0.09 (0.04, 0.14)	0.03 (0.01, 0.05)
Non-Hispanic Black	-0.02 (-0.08, 0.03)	-0.07 (-0.09, -0.04)
Hispanic	-0.06 (-0.13, 0.01)	-0.02 (-0.04, 0.003)
Other	0.08 (0.01, 0.15)	0.02 (-0.00, 0.05)

Data in the table are adjusted for calendar period of initiation (for 2011-2018 analysis), age, sex, antihypertensive medication class initiated during the follow-up period, antihypertensive medication regimen initiated during the follow-up period (single class, multiple classes with multiple pills, and fixed-dosed combination therapy), initiated with a 90-day fill, copay-per-day of supply, prevalent conditions, newly documented conditions, and Medicare Part D coverage.
Non-persistence was defined as not having antihypertensive medication available to take in the last 90 days of the follow-up period.
Abbreviations: PDC, interval-based proportion of days covered
