# Supplementary material for: Antihypertensive medication persistence and adherence among non-Hispanic Asian US patients with hypertension and fee-for-service Medicare health insurance
Source: PLoS One. 2024 Mar 20;19(3):e0300372. doi: 10.1371/journal.pone.0300372 (PMC10954118; doi:10.1371/journal.pone.0300372)
Supplement: S9 Table — (PDF) [file pone.0300372.s010.pdf]

S9 Table. Adjusted risk ratios for non-persistence associated with beneficiary characteristics within race/ethnicity groups.

	Race/ethnicity				
	Non-Hispanic Asian	Non-Hispanic White	Non-Hispanic Black	Hispanic	Other
Calendar period of initiation					
2011-2012	1 (ref)	1 (ref)	1 (ref)	1 (ref)	1 (ref)
2013-2014	0.90 (0.74-1.09)	1.01 (0.96-1.05)	0.88 (0.77-0.99)	0.97 (0.85-1.10)	0.91 (0.66-1.25)
2015-2016	0.92 (0.76-1.11)	1.05 (1.00-1.10)	1.05 (0.93-1.19)	0.87 (0.76-1.01)	0.86 (0.64-1.15)
2017-2018	0.99 (0.82-1.19)	1.07 (1.02-1.11)	0.88 (0.77-1.00)	1.03 (0.90-1.18)	0.86 (0.64-1.15)
Age, years					
66 – 74	1 (ref)	1 (ref)	1 (ref)	1 (ref)	1 (ref)
75 – 84	1.08 (0.93-1.26)	1.04 (1.00-1.07)	0.98 (0.89-1.09)	1.03 (0.92-1.15)	1.15 (0.91-1.45)
85+	1.13 (0.92-1.39)	1.06 (1.01-1.11)	1.05 (0.92-1.20)	1.12 (0.97-1.29)	0.98 (0.65-1.47)
Female sex	0.96 (0.83-1.10)	0.91 (0.88-0.94)	0.89 (0.81-0.97)	0.88 (0.80-0.98)	0.91 (0.73-1.13)
Antihypertensive medication class					
Thiazide diuretic	1.20 (0.69-2.11)	0.85 (0.74-0.96)	0.89 (0.72-1.10)	0.86 (0.60-1.23)	0.74 (0.38-1.45)
ACE inhibitor	0.98 (0.58-1.65)	0.64 (0.56-0.72)	0.77 (0.63-0.93)	0.86 (0.61-1.19)	0.54 (0.28-1.03)
Angiotensin receptor blocker	0.96 (0.57-1.61)	0.57 (0.50-0.65)	0.72 (0.57-0.90)	0.76 (0.54-1.08)	0.65 (0.34-1.26)
Calcium Channel blocker	1.03 (0.62-1.71)	0.69 (0.61-0.79)	0.75 (0.62-0.90)	0.79 (0.57-1.10)	0.56 (0.31-1.03)
Beta blocker	0.95 (0.57-1.58)	0.72 (0.64-0.82)	0.81 (0.66-0.99)	0.86 (0.61-1.20)	0.66 (0.34-1.28)
Loop diuretic	1.44 (0.83-2.48)	1.13 (1.00-1.29)	1.16 (0.94-1.42)	1.09 (0.78-1.54)	0.83 (0.41-1.66)
Other	1.13 (0.66-1.94)	0.87 (0.77-0.99)	0.88 (0.72-1.08)	0.92 (0.64-1.31)	0.96 (0.48-1.94)
Antihypertensive medication pills					
Single class	1 (ref)	1 (ref)	1 (ref)	1 (ref)	1 (ref)
Multiple classes with multiple Pills	0.47 (0.25-0.91)	0.84 (0.72-0.97)	0.93 (0.72-1.19)	0.81 (0.53-1.24)	0.83 (0.35-2.00)
Fixed-dosed combination therapy*	0.55 (0.28-1.10)	1.04 (0.89-1.21)	0.89 (0.66-1.20)	1.07 (0.70-1.64)	1.34 (0.61-2.96)
Initiated with a 90-day fill	0.79 (0.67-0.93)	0.72 (0.70-0.75)	0.88 (0.79-0.98)	0.92 (0.82-1.03)	0.75 (0.59-0.95)
Copay-per-day of supply, $					
Quartile 1 (< $0.0366)	1 (ref)	1 (ref)	1 (ref)	1 (ref)	1 (ref)
Quartile 2 ($0.0367- $0.0926)	1.15 (0.97-1.38)	1.12 (1.07-1.17)	1.11 (0.99-1.25)	1.01 (0.89-1.14)	1.28 (0.97-1.68)
Quartile 3 ($0.0927 - $0.1833)	0.96 (0.78-1.19)	1.06 (1.01-1.11)	1.05 (0.92-1.21)	0.92 (0.78-1.08)	1.14 (0.83-1.57)

Quartile 4 (≥ $0.1834)	1.18 (0.95-1.48)	1.18 (1.12-1.24)	1.08 (0.93-1.24)	0.99 (0.83-1.18)	1.02 (0.72-1.43)
Prevalent conditions					
Diabetes	1.13 (0.97-1.31)	0.99 (0.95-1.02)	1.05 (0.95-1.16)	0.94 (0.85-1.04)	0.99 (0.78-1.25)
CVD	0.88 (0.74-1.05)	1.04 (1.00-1.07)	0.97 (0.86-1.08)	1.04 (0.92-1.17)	0.92 (0.71-1.20)
Heart failure	1.13 (0.79-1.61)	0.86 (0.80-0.91)	1.05 (0.89-1.24)	1.11 (0.91-1.37)	1.34 (0.86-2.07)
CKD	0.98 (0.81-1.19)	1.02 (0.97-1.06)	0.92 (0.82-1.04)	0.98 (0.86-1.12)	1.20 (0.92-1.56)
Depression	0.89 (0.71-1.10)	0.99 (0.96-1.03)	1.08 (0.96-1.22)	0.76 (0.67-0.87)	0.88 (0.66-1.16)
Serious fall injury	1.40 (0.91-2.15)	1.08 (0.99-1.17)	1.36 (1.00-1.85)	0.92 (0.66-1.27)	1.48 (0.81-2.72)
Polypharmacy	1.10 (0.94-1.30)	1.11 (1.07-1.15)	1.15 (1.03-1.28)	1.17 (1.04-1.30)	0.95 (0.73-1.23)
Following treatment initiation					
Newly documented diabetes	0.87 (0.48-1.60)	0.89 (0.76-1.05)	0.76 (0.51-1.13)	0.93 (0.62-1.40)	0.53 (0.15-1.86)
Newly documented CKD	1.40 (1.01-1.93)	0.94 (0.86-1.02)	0.78 (0.61-1.00)	0.74 (0.54-1.02)	0.91 (0.49-1.69)
Newly documented CVD	0.98 (0.69-1.39)	0.87 (0.80-0.95)	1.04 (0.83-1.30)	0.65 (0.48-0.89)	0.82 (0.45-1.47)
Newly documented depression	1.07 (0.73-1.59)	0.84 (0.77-0.92)	0.70 (0.53-0.92)	0.64 (0.48-0.86)	0.73 (0.42-1.28)
Serious fall injury	0.48 (0.07-3.52)	1.02 (0.88-1.18)	0.87 (0.40-1.88)	1.04 (0.62-1.75)	4.11 (2.49-6.79)
Medicare Part D coverage gap	0.81 (0.68-0.98)	0.86 (0.83-0.90)	0.65 (0.56-0.76)	0.82 (0.72-0.93)	0.75 (0.56-1.01)

Data in the table are risk ratios (95% confidence intervals) from regression models including all of the variables listed in the left column.

*Fixed-dosed combination therapy is defined as initiating treatment with a single pill containing 2 or more antihypertensive classes. If a patient was prescribed fixed-dose combination therapy and an additional antihypertensive medication in another pill, the patient was categorized as taking fixed-dose combination therapy.

Abbreviations: ACE, angiotensin-converting enzyme; CVD, cardiovascular disease; CKD, chronic kidney disease.
