# Supplementary material for: Antihypertensive medication persistence and adherence among non-Hispanic Asian US patients with hypertension and fee-for-service Medicare health insurance
Source: PLoS One. 2024 Mar 20;19(3):e0300372. doi: 10.1371/journal.pone.0300372 (PMC10954118; doi:10.1371/journal.pone.0300372)
Supplement: S10 Table — (PDF) [file pone.0300372.s011.pdf]

S10 Table. Race/ethnicity-specific adjusted risk ratios for having low adherence among all beneficiaries initiating antihypertensive medication.

	Race/ethnicity				
	Non-Hispanic Asian	Non-Hispanic White	Non-Hispanic Black	Hispanic	Other
Calendar period of initiation					
2011-2012	1 (ref)	1 (ref)	1 (ref)	1 (ref)	1 (ref)
2013-2014	0.94 (0.85-1.04)	0.98 (0.96-1.00)	0.92 (0.86-0.97)	0.93 (0.87-0.99)	0.99 (0.84-1.16)
2015-2016	0.92 (0.83-1.02)	0.97 (0.95-1.00)	0.98 (0.92-1.04)	0.89 (0.83-0.96)	0.93 (0.79-1.09)
2017-2018	0.92 (0.82-1.02)	0.94 (0.92-0.97)	0.94 (0.88-1.00)	0.90 (0.84-0.96)	0.88 (0.75-1.03)
Age, years					
66 – 74	1 (ref)	1 (ref)	1 (ref)	1 (ref)	1 (ref)
75 – 84	0.98 (0.90-1.07)	1.00 (0.98-1.02)	0.96 (0.91-1.01)	1.02 (0.96-1.07)	1.22 (1.09-1.38)
85+	1.01 (0.90-1.13)	0.98 (0.96-1.01)	0.98 (0.92-1.05)	1.02 (0.94-1.10)	1.20 (0.99-1.46)
Female sex	1.03 (0.95-1.11)	0.97 (0.95-0.99)	0.96 (0.92-1.01)	0.91 (0.87-0.96)	0.98 (0.87-1.09)
Antihypertensive medication class					
Thiazide diuretic	1.16 (0.91-1.48)	0.84 (0.78-0.91)	0.96 (0.86-1.06)	0.95 (0.81-1.13)	0.82 (0.59-1.15)
ACE inhibitor	1.06 (0.85-1.31)	0.71 (0.66-0.76)	0.88 (0.80-0.96)	0.89 (0.76-1.04)	0.75 (0.55-1.02)
Angiotensin receptor blocker	1.04 (0.84-1.29)	0.70 (0.65-0.75)	0.85 (0.76-0.95)	0.93 (0.80-1.09)	0.77 (0.55-1.06)
Calcium Channel blocker	1.08 (0.88-1.33)	0.73 (0.68-0.78)	0.87 (0.80-0.96)	0.94 (0.81-1.09)	0.69 (0.50-0.95)
Beta blocker	1.13 (0.91-1.39)	0.77 (0.72-0.83)	0.87 (0.79-0.96)	0.88 (0.76-1.03)	0.81 (0.59-1.12)
Loop diuretic	1.44 (1.14-1.81)	1.11 (1.03-1.19)	1.10 (0.99-1.21)	1.03 (0.87-1.21)	0.83 (0.58-1.17)
Other	1.11 (0.88-1.39)	0.90 (0.84-0.97)	0.99 (0.90-1.09)	0.98 (0.83-1.16)	0.93 (0.67-1.28)
Antihypertensive medication pills					
Single class	1 (ref)	1 (ref)	1 (ref)	1 (ref)	1 (ref)
Multiple classes with multiple pills	0.74 (0.56-0.98)	0.91 (0.84-0.99)	0.87 (0.77-0.99)	0.83 (0.69-1.01)	1.13 (0.76-1.67)
Fixed-dosed combination therapy*	0.84 (0.62-1.13)	1.22 (1.12-1.33)	1.05 (0.92-1.21)	1.09 (0.90-1.32)	1.02 (0.66-1.58)
Initiated with a 90-day fill	0.85 (0.77-0.93)	0.83 (0.81-0.85)	0.95 (0.90-1.00)	0.89 (0.84-0.95)	0.89 (0.78-1.00)
Copay-per-day of supply, $					
Quartile 1 (< $0.0366)	1 (ref)	1 (ref)	1 (ref)	1 (ref)	1 (ref)
Quartile 2 ($0.0367- $0.0926	1.04 (0.94-1.15)	1.11 (1.08-1.14)	1.18 (1.11-1.25)	1.01 (0.95-1.08)	1.11 (0.97-1.28)

Quartile 3 ($0.0927 - $0.1833)	0.94 (0.84-1.06)	1.07 (1.04-1.10)	1.09 (1.01-1.17)	0.95 (0.87-1.03)	1.05 (0.89-1.24)
Quartile 4 (≥ $0.1834)	1.03 (0.90-1.16)	1.15 (1.12-1.19)	1.14 (1.06-1.23)	0.90 (0.82-0.99)	1.01 (0.85-1.20)
Prevalent conditions					
Diabetes	1.06 (0.98-1.16)	1.03 (1.01-1.05)	1.02 (0.97-1.07)	1.01 (0.96-1.07)	1.10 (0.98-1.25)
CVD	1.06 (0.97-1.17)	1.04 (1.02-1.06)	1.01 (0.96-1.07)	1.04 (0.98-1.11)	0.94 (0.82-1.08)
Heart failure	0.93 (0.76-1.15)	0.89 (0.85-0.92)	1.07 (0.99-1.16)	0.94 (0.84-1.06)	1.07 (0.84-1.36)
CKD	0.93 (0.83-1.04)	1.00 (0.98-1.03)	1.00 (0.95-1.06)	1.00 (0.93-1.07)	1.03 (0.89-1.19)
Depression	0.95 (0.85-1.07)	0.97 (0.95-0.99)	0.99 (0.93-1.06)	0.87 (0.81-0.93)	1.10 (0.96-1.26)
Serious fall injury	1.39 (1.16-1.66)	1.03 (0.98-1.08)	1.09 (0.91-1.30)	0.88 (0.74-1.05)	1.01 (0.69-1.49)
Polypharmacy	1.00 (0.91-1.10)	1.12 (1.10-1.14)	1.08 (1.02-1.14)	1.13 (1.06-1.19)	0.96 (0.84-1.10)
Following treatment initiation					
Newly documented diabetes	1.16 (0.89-1.51)	0.92 (0.83-1.01)	0.91 (0.76-1.08)	0.93 (0.75-1.16)	1.13 (0.76-1.69)
Newly documented CKD	1.23 (1.04-1.46)	1.01 (0.97-1.06)	0.98 (0.88-1.09)	0.87 (0.75-1.01)	0.91 (0.65-1.26)
Newly documented CVD	1.15 (0.96-1.36)	1.00 (0.95-1.05)	1.02 (0.92-1.14)	0.83 (0.71-0.95)	0.86 (0.63-1.18)
Newly documented depression	0.84 (0.64-1.09)	0.91 (0.87-0.96)	1.00 (0.89-1.11)	0.85 (0.75-0.98)	1.05 (0.81-1.36)
Serious fall injury	1.05 (0.59-1.87)	1.20 (1.11-1.29)	1.16 (0.87-1.55)	1.27 (1.04-1.54)	2.08 (1.79-2.41)
Medicare Part D coverage gap	0.79 (0.71-0.88)	0.85 (0.83-0.87)	0.73 (0.68-0.79)	0.79 (0.74-0.85)	0.76 (0.65-0.90)

Data in the table are risk ratios (95% confidence intervals) from regression models including all of the variables listed in the left column

*Fix-dosed combination therapy is defined as initiating treatment with a single pill containing 2 or more antihypertensive classes. If a patient was prescribed fixed-dose combination therapy and an additional antihypertensive medication in another pill, the patient was categorized as taking fixed-dose combination therapy.

Abbreviations: ACE, angiotensin-converting enzyme; CVD, cardiovascular disease; CKD, chronic kidney disease.
