# Supplementary material for: Antihypertensive medication persistence and adherence among non-Hispanic Asian US patients with hypertension and fee-for-service Medicare health insurance
Source: PLoS One. 2024 Mar 20;19(3):e0300372. doi: 10.1371/journal.pone.0300372 (PMC10954118; doi:10.1371/journal.pone.0300372)
Supplement: S11 Table — (PDF) [file pone.0300372.s012.pdf]

**S11 Table. Race/ethnicity-specific adjusted risk ratios for low adherence among beneficiaries who were persistent to their antihypertensive medication.**

| | Race/ethnicity | | | | |
|---|---|---|---|---|---|
| | Non-Hispanic Asian | Non-Hispanic White | Non-Hispanic Black | Hispanic | Other |
| Calendar period of initiation | | | | | |
| 2011-2012 | 1 (ref) | 1 (ref) | 1 (ref) | 1 (ref) | 1 (ref) |
| 2013-2014 | 0.95 (0.80-1.12) | 0.96 (0.93-1.00) | 0.91 (0.83-1.00) | 0.89 (0.80-0.99) | 1.01 (0.79-1.28) |
| 2015-2016 | 0.89 (0.75-1.06) | 0.92 (0.88-0.96) | 0.94 (0.85-1.03) | 0.86 (0.77-0.96) | 0.92 (0.73-1.16) |
| 2017-2018 | 0.84 (0.70-1.01) | 0.85 (0.82-0.89) | 0.94 (0.86-1.03) | 0.80 (0.71-0.90) | 0.83 (0.66-1.05) |
| Age, years | | | | | |
| 66 – 74 | 1 (ref) | 1 (ref) | 1 (ref) | 1 (ref) | 1 (ref) |
| 75 – 84 | 0.91 (0.79-1.05) | 0.98 (0.95-1.02) | 0.95 (0.88-1.02) | 1.00 (0.92-1.10) | 1.34 (1.12-1.61) |
| 85+ | 0.93 (0.77-1.14) | 0.93 (0.89-0.97) | 0.96 (0.86-1.06) | 0.98 (0.86-1.12) | 1.39 (1.05-1.84) |
| Female sex | 1.08 (0.95-1.23) | 1.01 (0.98-1.04) | 0.97 (0.91-1.05) | 0.90 (0.83-0.98) | 0.99 (0.83-1.17) |
| Antihypertensive medication class | | | | | |
| Thiazide diuretic | 1.18 (0.84-1.65) | 0.82 (0.73-0.91) | 0.96 (0.83-1.11) | 0.98 (0.77-1.25) | 0.82 (0.53-1.25) |
| ACE inhibitor | 1.09 (0.82-1.46) | 0.70 (0.64-0.77) | 0.88 (0.77-1.00) | 0.87 (0.70-1.09) | 0.81 (0.54-1.21) |
| Angiotensin receptor blocker | 1.08 (0.82-1.43) | 0.72 (0.66-0.80) | 0.85 (0.73-0.99) | 1.00 (0.79-1.26) | 0.79 (0.52-1.20) |
| Calcium Channel blocker | 1.11 (0.85-1.44) | 0.69 (0.63-0.76) | 0.89 (0.79-1.01) | 1.00 (0.81-1.25) | 0.72 (0.48-1.07) |
| Beta blocker | 1.24 (0.95-1.62) | 0.76 (0.69-0.84) | 0.85 (0.74-0.97) | 0.85 (0.67-1.07) | 0.86 (0.57-1.30) |
| Loop diuretic | 1.63 (1.16-2.27) | 1.19 (1.08-1.32) | 1.12 (0.97-1.30) | 1.04 (0.81-1.33) | 0.80 (0.49-1.32) |
| Other | 1.13 (0.82-1.56) | 0.91 (0.83-1.01) | 1.02 (0.90-1.16) | 1.02 (0.80-1.32) | 0.88 (0.58-1.33) |
| Antihypertensive medication pills | | | | | |
| Single class | 1 (ref) | 1 (ref) | 1 (ref) | 1 (ref) | 1 (ref) |
| Multiple classes with multiple pills | 0.84 (0.59-1.19) | 0.92 (0.82-1.03) | 0.82 (0.69-0.98) | 0.80 (0.60-1.07) | 1.25 (0.76-2.05) |
| Fixed-dosed combination therapy* | 0.99 (0.66-1.49) | 1.37 (1.21-1.55) | 1.13 (0.94-1.37) | 1.12 (0.83-1.50) | 0.90 (0.50-1.63) |
| Initiated with a 90-day fill | 0.83 (0.72-0.95) | 0.86 (0.84-0.89) | 0.97 (0.89-1.05) | 0.83 (0.75-0.92) | 0.93 (0.78-1.12) |

| | | | | | |
|---|---|---|---|---|---|
| Copay-per-day of supply, $ | | | | | |
| Quartile 1 (< $0.0366) | 1 (ref) | 1 (ref) | 1 (ref) | 1 (ref) | 1 (ref) |
| Quartile 2 ($0.0367- $0.0926 | 0.99 (0.84-1.16) | 1.14 (1.09-1.19) | 1.27 (1.15-1.39) | 1.01 (0.91-1.12) | 1.07 (0.87-1.31) |
| Quartile 3 ($0.0927 - $0.1833) | 0.92 (0.76-1.10) | 1.10 (1.05-1.15) | 1.14 (1.02-1.27) | 0.94 (0.82-1.07) | 1.02 (0.81-1.30) |
| Quartile 4 (≥ $0.1834) | 0.94 (0.77-1.15) | 1.19 (1.14-1.24) | 1.22 (1.10-1.36) | 0.82 (0.70-0.95) | 1.00 (0.77-1.29) |
| Prevalent conditions | | | | | |
| Diabetes | 1.05 (0.91-1.21) | 1.06 (1.03-1.10) | 1.01 (0.94-1.09) | 1.05 (0.96-1.14) | 1.21 (1.01-1.44) |
| CVD | 1.22 (1.05-1.41) | 1.05 (1.02-1.09) | 1.03 (0.94-1.12) | 1.06 (0.96-1.17) | 0.94 (0.76-1.15) |
| Heart failure | 0.77 (0.52-1.13) | 0.85 (0.80-0.91) | 1.10 (0.97-1.24) | 0.81 (0.65-1.00) | 0.97 (0.65-1.47) |
| CKD | 0.87 (0.72-1.05) | 0.99 (0.95-1.03) | 1.03 (0.95-1.13) | 1.00 (0.89-1.12) | 0.94 (0.75-1.18) |
| Depression | 0.96 (0.79-1.16) | 0.95 (0.92-0.98) | 0.95 (0.86-1.05) | 0.87 (0.79-0.97) | 1.24 (1.01-1.50) |
| Serious fall injury | 1.64 (1.22-2.22) | 1.00 (0.92-1.09) | 0.99 (0.72-1.36) | 0.81 (0.61-1.09) | 0.77 (0.36-1.65) |
| Polypharmacy | 0.96 (0.82-1.12) | 1.17 (1.13-1.20) | 1.08 (0.99-1.17) | 1.16 (1.06-1.27) | 0.95 (0.78-1.16) |
| Following treatment initiation | | | | | |
| Newly documented diabetes | 1.42 (0.96-2.09) | 0.91 (0.79-1.06) | 0.94 (0.74-1.19) | 0.91 (0.65-1.29) | 1.52 (0.91-2.55) |
| Newly documented CKD | 1.25 (0.92-1.68) | 1.06 (0.98-1.13) | 1.05 (0.90-1.23) | 0.88 (0.71-1.10) | 0.90 (0.55-1.46) |
| Newly documented CVD | 1.33 (1.00-1.75) | 1.07 (1.00-1.15) | 1.02 (0.86-1.21) | 0.87 (0.71-1.07) | 0.89 (0.55-1.44) |
| Newly documented depression | 0.63 (0.38-1.03) | 0.94 (0.87-1.01) | 1.11 (0.96-1.29) | 0.91 (0.75-1.10) | 1.20 (0.85-1.69) |
| Serious fall injury | 1.30 (0.66-2.57) | 1.38 (1.23-1.55) | 1.32 (0.89-1.95) | 1.52 (1.12-2.07) | 4.49 (2.81-7.20) |
| Medicare Part D coverage gap | 0.70 (0.59-0.84) | 0.81 (0.78-0.84) | 0.70 (0.63-0.79) | 0.72 (0.64-0.80) | 0.73 (0.57-0.92) |

Data in the table are risk ratios (95% confidence intervals) from regression models including all of the variables listed in the left column

*Fix-dosed combination therapy is defined as initiating treatment with a single pill containing 2 or more antihypertensive classes. If a patient was prescribed fixed-dose combination therapy and an additional antihypertensive medication in another pill, the patient was categorized as taking fixed-dose combination therapy.

Abbreviations: ACE, angiotensin-converting enzyme; CVD, cardiovascular disease; CKD, chronic kidney disease.
